# Direct-acting antiviral agent efficacy and safety in renal transplant recipients with chronic hepatitis C virus infection: A PRISMA-compliant study

**DOI:** 10.1097/MD.0000000000007568

**Published:** 2017-07-28

**Authors:** Keliang Chen, Pei Lu, Rijin Song, Jiexiu Zhang, Rongzhen Tao, Zijie Wang, Wei Zhang, Min Gu

**Affiliations:** Department of Urology, First Affiliated Hospital with Nanjing Medical University, Nanjing, China.

**Keywords:** adverse event, direct-acting antivirals, hepatitis C infection, renal transplant recipients, sustained virological response

## Abstract

**Background:**

The efficacy and safety of direct-acting antivirals (DAAs) for treating hepatitis C virus (HCV)-infected renal transplant recipients (RTRs) has not been determined.

**Methods:**

We searched PubMed, Embase, and the Cochrane Central Register of Controlled Trials and assessed the quality of eligible studies using the Joanna Briggs Institute scale. DAA efficacy and safety were assessed using standard mean difference (SMD) with 95% confidence intervals (95%CIs).

**Results:**

Six studies (360 RTRs) were included. Two hundred thirty six RTRs (98.3%) achieved sustained virological response within 12 weeks; HCV infection was cleared in 239 RTRs after 24-week treatment. Liver function differed significantly pre- and posttreatment (alanine aminotransferase, SMD: 0.96, 95%CIs: 0.65, 1.26; aspartate aminotransferase, SMD: 0.89, 95%CIs: 0.60, 1.18); allograft function pre- and posttreatment was not statistically different (serum creatinine, SMD: −0.13, 95%CIs: −0.38, 0.12; estimated glomerular filtration rate, SMD: 0.20, 95%CIs: −0.11, 0.51). General symptoms (fatigue nausea dizziness or headache) were the most common adverse events (AEs) (39.3%). Severe AEs, that is, anemia, portal vein thrombosis, and streptococcus bacteraemia and pneumonia, were present in 1.1%, 0.6%, and 1.1% of RTRs, respectively.

**Conclusion:**

Our findings suggest that DAAs are highly efficacious and safe for treating HCV-infected RTRs and without significant AE.

## 1 Introduction

Due to its high incidence, chronic hepatitis C virus (HCV) infection remains troublesome worldwide, and indirectly presents a considerable challenge to renal transplant recipients (RTRs). Nearly, 1.8% to 8% of RTRs in the developed countries are infected with HCV.^[1,2]^ HCV-infected RTRs are significantly more likely to have other infections, new-onset diabetes mellitus, cardiovascular diseases, and liver fibrosis compared with other RTRs.^[3,4]^ In addition, the development of HCV viremia and liver fibrosis can be accelerated after long-term immunosuppressive therapy, which contributes to the poor prognosis of RTRs with HCV infections post-transplantation.^[5]^ On the other hand, despite these risks, the survival of HCV-infected RTRs is significantly higher when compared with RTRs who depend on the maintenance treatment of hemodialysis.^[6]^

To date, few anti-HCV therapies eliminate HCV efficiently and safely. Interferon-α (IFN-α), ribavirin (RBV), and protease inhibitor (PI)-based therapies have been established as the major treatment options for HCV infection.^[7–9]^ Nevertheless, IFN-α is associated with poor sustained virological response (SVR) rates (13–43%) and is contraindicated due to the high incidence of adverse events (AEs).^[10,11]^ Monotherapy with RBV did not affect the clearance of HCV loads.^[12–14]^ In addition, the option of PI-based treatments is limited after kidney transplantation due to drug–drug interactions with calcineurin inhibitors, and severe AEs.^[15–18]^

The novel, all-oral direct-acting antivirals (DAAs) were recently identified as being significantly efficient for treating HCV infections in RTRs.^[19–23]^ HCV RNA is translated into a long polyprotein that includes NS5A protein, NS5B polymerase, and NS3/4A protease, which DAAs target. Currently, 5 DAAs are mainly administered in HCV infection; Table 1 lists the related mechanisms.^[24]^ Among RTRs who received the basic treatment, the combination administration of at least 2 different classes of DAAs achieved an SVR rate at 12 weeks after completing therapy (SVR12) of over 90%.^[20,25,26]^ Moreover, DAAs were effective for cirrhotic patients: the SVR rate was approximately 85.9% among cirrhotic patients who were Child-Pugh A and 82.2% for Child-Pugh B/C patients.^[27]^ However, systematic evaluation of DAA efficacy and safety for treating RTRs with HCV infections following kidney transplantation is lacking.

**Table 1:**
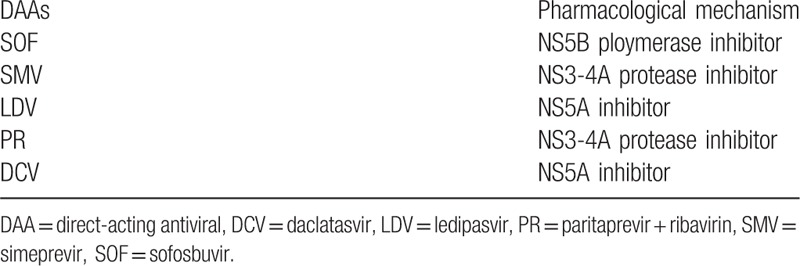
The main types of DAAs and their mechanisms.

In this study, we performed a comprehensive systematic review on DAA efficacy and safety in clearing HCV in RTRs. Then, the pooled data of selected articles were used to assess the influence of DAA therapy on RTRs.

## 2 Methods

### 2.1 Literature search

Two reviewers (KLC and PL) reviewed studies on DAA efficacy and safety in clearing HCV in RTRs independently. PubMed, Embase, and the Cochrane Central Register of Controlled Trials were comprehensively searched until February 1, 2017. The following key word combination was used: (“HCV or hepatitis C virus” and “DAAs or direct-acting antiviral agents,” and MeSH items “kidney transplantation”). The reference lists of eligible studies were also checked. The first or corresponding author of each study was contacted when the results were unclear or when sufficient data were not reported. If more than 1 article with the same content was published, we selected the most complete article.

### 2.2 Inclusion and exclusion criteria

The inclusion criteria were: case–control trial or cohort study designed to investigate DAA efficacy and safety in clearing HCV in RTRs, availability of relevant data focused on DAA efficacy and safety, and all RTRs in eligible studies were over 18 years old. Two authors (KLC and ZJW) assessed and selected trials for the final analysis according to these criteria independently; disagreements were resolved by consensus of a third author (PL). Studies with insufficient data for pooling were excluded.

### 2.3 Data extraction and quality assessment

Two investigators (KLC and ZJW) extracted data from all potentially relevant studies independently. The following characteristics were recorded: first author's name, year of publication, ethnicity, and number of included patients, number of male and female patients, immunosuppressive protocols, DAA protocols, and results of DAA efficacy and safety. Missing data were also examined by contacting the first or corresponding author. Conflicting evaluations were resolved by discussion.

The Joanna Briggs Institute scale, which contains 10 items; each item is judged “YES,” “NO” or “UNCLEAR,”^[28]^ was used to assess the quality of all included studies. A score of 0 to 20 was assigned to each study, with 0 being the lowest and 20 being the best quality.

### 2.4 Statistical analysis

The pooled data were used to assess DAA efficacy and safety in clearing HCV infection in RTRs by the standard mean difference (SMD) with 95% confidence intervals (95%CIs). *P* < .05 was considered statistically significant. Heterogeneity among trials was determined by *I*^2^, which was defined as 100% × (*Q* − *df*)/*Q*, where *Q* is Cochran heterogeneity statistic and *df* is the degrees of freedom, using a fixed-effect model set at low statistical inconsistency (*I*^2^ < 50%); otherwise, we used a random-effects model, which is better adapted to clinical and statistical variations. All statistical analyses were performed using STATA (release 12.0, College Station, TX).

We didn’t need to obtain ethical approval or informed consent because our data were extracted from previous studies. Nevertheless, the included studies in our review did get patient consent and each study was approved by an ethics committee.

## 3 Results

### 3.1 Study selection and basic characteristics

**Figure 1:**
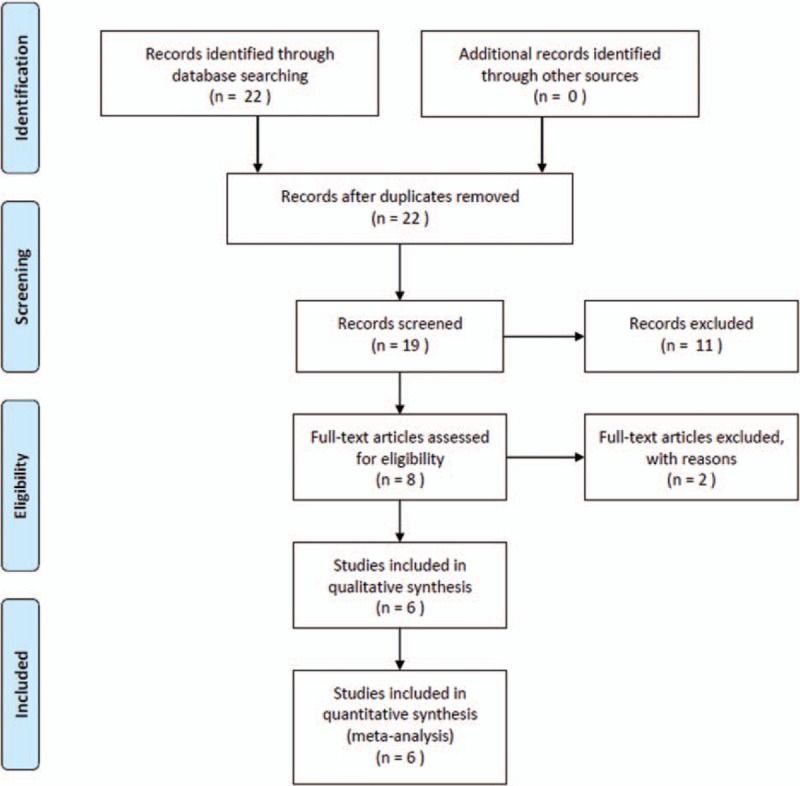
Flow chart of selected articles.

A total of 22 articles were identified after the comprehensive literature research. Three duplicates were removed and 19 articles remained. Eight studies were included after reviewing the titles and abstracts, of which 2 were excluded due to insufficient data for pooling. An eventual 6 full-text articles involving 360 RTRs were eligible for the quantitative synthesis (Fig. 1).^[29–34]^

Table 2 summarizes the baseline patient demographic and clinical characteristics. Except the research studied by Roth et al^[34]^ included both Caucasian and Negroes, all patients were Caucasian and the majority were men; the mean age was approximately 56 years. The studies described 9 DAA therapies: sofosbuvir (SOF) and simeprevir (SMV) (n = 32); SOF and ledipasvir (LDV) (n = 45); SOF and daclatasvir (DCV) (n = 21); SOF and RBV (n = 15); SOF and paritaprevir + RBV (PR) (n = 2); SMV and DCV (n = 1); SOF, SMV, and RBV (n = 4); SOF, LDV, and RBV (n = 5); (9) grazoprevir (GAV) and elbasvir (EBV) (n = 235).

**Table 2:**
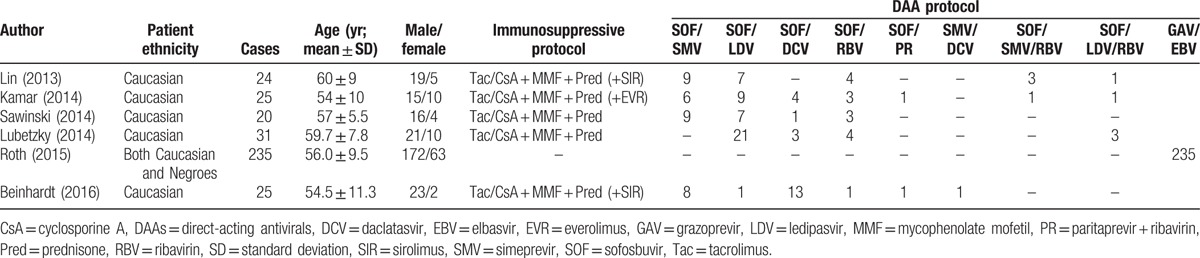
Basic characteristics of eligible studies.

### 3.2 DAA efficacy

Almost all patients completed DAA treatment and were followed for at least 12 weeks after treatment. Only 1 patient achieved SVR4 but died prior to the SVR12 checkpoint due to treatment-unrelated causes. This patient was excluded from the primary endpoint analysis.^[30]^ Kamar et al^[31]^ reported HCV RNA concentrations of 6.41 ± 0.63 log IU/mL at baseline, which decreased to 0.23 ± 0.61 log IU/mL at week 4 after DAA treatment was started. Sawinski et al^[33]^ reported the same trend: the viral load at therapy initiation was 6.5 ± 0.61 log IU/mL and decreased to 0.57 ± 1.0 log IU/mL after 4 weeks of therapy. In Roth study, 235 patients received study drug, 224 were assigned to the immediate treatment group (n = 111) or deferred treatment group (n = 113), and an additional 11 patients were assigned to the intensive pharmacokinetic treatment group. Deferred treatment group were of no statistical significance because they started treatment after 12 weeks. Of the 122 patients in the immediate treatment and intensive pharmacokinetic population, 6 were excluded for reasons of death, lost to follow-up, noncompliance, and so on. Of the remaining 116 patients, 115 (115/116, 99%) achieved SVR12.^[34]^ Overall, the virus was cleared in 236 patients (236/240, 98.3%) within 12 weeks. Only 3 RTRs (2.4%) received a 24-week course of DAAs, 1 patient relapsed after the end of treatment and the virus was cleared in 239 patients after the 24-week treatment (Table 3).

**Table 3:**
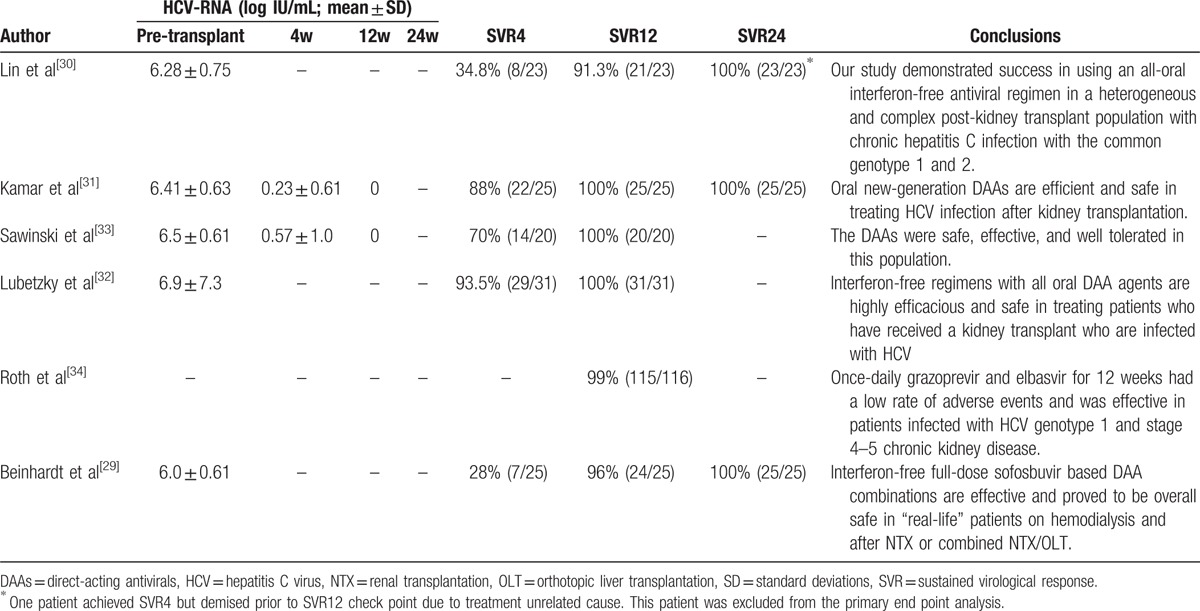
Systematic review of efficacy of DAAs in renal transplant recipients.

### 3.3 DAA safety and tolerance

**Figure 2:**
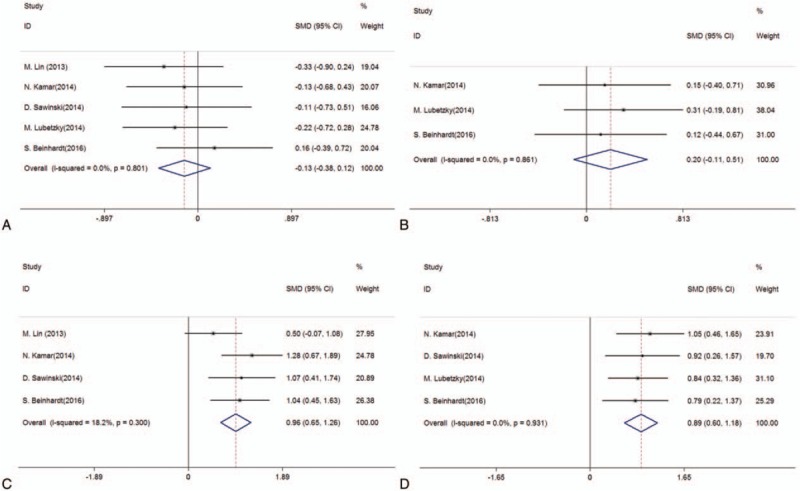
Forest plots from meta-analysis of DAA therapy on serum creatinine (A), eGFR (B), ALT (C), and AST (D) of RTRs with HCV infection. ALT = alanine aminotransferase; AST = aspartate aminotransferase; DAAs = direct-acting antivirals; eGFR, estimated glomerular filtration rate; HCV =  hepatitis C virus; RTRs = renal transplant recipients.

Liver function was significantly improved after DAA therapy, where alanine aminotransferase (ALT) and aspartate aminotransferase (AST) were both remarkably decreased after treatment (ALT, SMD: 0.96, 95%CIs: 0.65, 1.26, *P* < .001; AST, SMD: 0.89, 95%CIs: 0.60, 1.18, *P* < .001; Table 4 and Fig. 2). Moreover, allograft function was not significantly different pre- and post-DAA therapy (serum creatinine [Scr], SMD: −0.13, 95%CIs: −0.38, 0.12, *P* = .31; estimated glomerular filtration rate [eGFR], SMD: 0.20, 95%CIs: −0.11, 0.51, *P* = .20; Table 4 and Fig. 2). Roth's article didn’t describe the specific values of liver and renal function, but it reported that the ALT and AST were risen more among patients without treatment than the treatment group and there was no significant change in mean eGFR or Scr after treatment.^[34]^

**Table 4:**
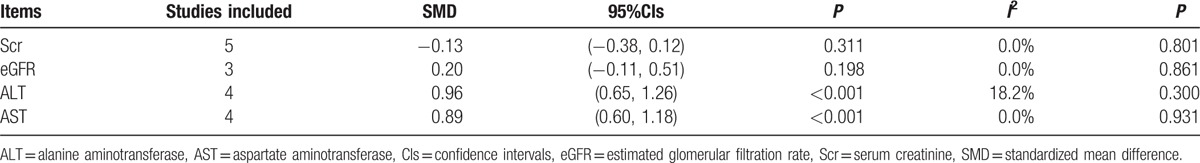
Meta-analysis of pooled results of safety of DAAs in renal transplant recipients.

The common AEs were general symptoms (fatigue nausea dizziness or headache, 39.3%, 137/349), gastrointestinal symptoms (gastrointestinal bleeding or diarrhoea, 7.2%, 25/349), unstable blood pressure (1.1%, 4/349), and skin problems (photosensitivity or rash, 0.9%, 3/349). There were severe AEs, that is, anemia, portal vein thrombosis, and streptococcus bacteraemia, and pneumonia, in 1.1%, 0.6%, and 1.1% of patients, respectively. Four patients had anemia, 1 of whom required blood transfusion due to symptomatic anemia.^[33]^ After completion of the 24-week treatment, 2 patients were treated for pneumonia and recovered without requiring modification of the DAA doses.^[29,32]^ Two patients had portal vein thrombosis and streptococcus bacteraemia, and were treated with antibiotics and warfarin, and the complications were subsequently completely resolved (Table 5).^[29,30]^

**Table 5:**
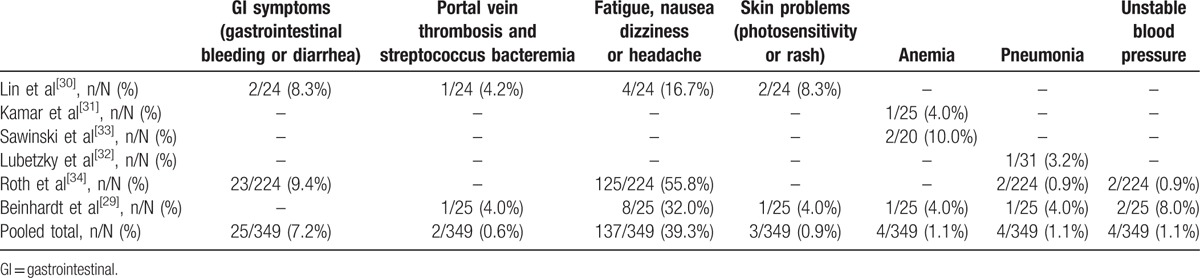
Meta-analysis of adverse events in renal transplant recipients.

## 4 Discussion

In RTRs, HCV infection dramatically increases the risk of death, which is associated with cardiovascular disease, infections, hepatic fibrosis, posttransplant diabetes mellitus, and cancer. Moreover, the situation will continue to worsen as RTRs receive immunosuppressive therapy.^[3–5]^ Previous anti-HCV infection therapies had comparatively high rates of treatment-limiting AEs and low rates of virological response for HCV-infected RTRs.^[7–18]^ Therefore, there is a need for effective and safe therapeutic options to cure HCV infection effectively. Very recently, the successful application of DAAs for treating HCV infection in RTRs was reported.^[31]^ Accordingly, we pooled the eligible data and analyzed their applicability in this study.

HCV RNA is translated into a long polyprotein that contains 3 structural proteins (C, E1, E2) and 7 non-structural proteins (p7, NS2, NS3, NS4A, NS4B, NS5A, NS5B). DAAs, which are inhibitors of NS3/4A protease, NS5A and NS5B polymerase, block HCV RNA transcription, and then remove the virus efficiently. SOF is a polymerase inhibitor of the HCV NS5B protein; LDV and DCV are pangenotypic NS5A inhibitors; SMV and paritaprevir are NS3/4A protease inhibitors.^[24]^ The combination of at least 2 different classes of DAAs is associated with high SVR and is the gold standard for treating HCV infection.^[20,25,26]^ In the 6 articles included in the present study, where a total 360 HCV-infected RTRs received new-generation DAAs, treatment efficacy was highly significant. Five patients were excluded from the study because of treatment-unrelated death prior to the SVR12 checkpoint, 113 patients were excluded because they were assigned to the deferred treatment group who started treatment after 12 weeks, another 2 were excluded for reasons of lost to follow-up and noncompliance.^[35]^ The virus was cleared in 239 RTRs after the 24-week treatment. The SVR12 rate was up to 98.3%, meaning only 4 patients did not complete treatment within 12 weeks. Therefore, the virus was cleared from almost all patients after 12 weeks and the efficacy after 4 weeks of treatment was good (SVR4 = 64.5%). The excellent efficacy of DAAs on the clearance of HCV could have a great impact on the selection of donor allograft. In our kidney transplantation center, HCV positive allograft could only be paired to recipients with HCV infection. With the introduction of DAAs, there is potential possibility that HCV positive allograft could be transplanted to recipients without HCV infection with the administration of DAAs, which was indicated as Coilly and Samuel.^[35]^ Moreover, several authors have reported the effectiveness of DAAs for treating HCV infection after liver transplantation. Kwo et al^[36]^ first studied 34 HCV-infected liver transplant recipients who received DAAs for 24 weeks and reported an SVR12 rate of up to 97%. Saab et al^[37]^ reported that the SVR12 rate was 93%. A meta-analysis that included 9 studies involving 325 post-liver transplant recipients reported a pooled rate of SVR12 of 88% after DAA treatment.^[38]^ Interestingly, the COSMOS study reported that the SVR12 rate in non-transplant patients was 90% to 94%, where the same DAAs as the meta-analysis mentioned above were used.^[20]^ Overall, DAAs achieve remarkable results in kidney transplantation, liver transplantation, or non-transplantation patients with HCV infection, which could significantly contribute the clearance of HCV among patients.

The biggest challenge of HCV therapies is safety of these agents. In the present, our results demonstrate that renal function (serum creatinine and eGFR) remained stable after DAA treatment. Lubetzky et al^[33]^ studied 20 HCV-infected patients without DAA treatment and found no difference in graft function. These findings indicate that DAA therapy does not affect renal function, and as the virus is removed, the glomerulonephritis causes is relieved, which greatly increases the success rate of the kidney transplant.^[39]^ Apart from its effect on the kidney, HCV infection may lead to end-stage liver disease, such as cirrhosis, and hepatocellular carcinoma.^[39]^ In addition, immunosuppressive therapy may aggravate the action of HCV and accelerate fibrosis progression in liver transplant recipients.^[5]^ Theoretically, liver function would improve greatly after HCV clearance by DAAs; our results, which show that both ALT and AST were decreased significantly after DAA treatment, verify the theory.

In terms of AEs, there were no severe complications, including serious infections that required cessation of therapy or warranted hospitalization during treatment. The most common AEs were general symptoms (fatigue nausea dizziness or headache, 39.3%, 137/349), gastrointestinal symptoms (gastrointestinal bleeding or diarrhoea, 7.2%, 25/349), and unstable blood pressure (1.1%, 4/349). The severe AEs were anemia, portal vein thrombosis, and streptococcus bacteraemia and pneumonia. One of the 4 patients with anemia required blood transfusion.^[33]^ After the completion of the 24-week treatment, 2 patients were treated for pneumonia and their symptoms improved without needing modification of DAA dose.^[37,33]^ Two patients with portal vein thrombosis and streptococcus bacteraemia were treated with antibiotic and warfarin, and the complications were subsequently completely resolved.^[29,30]^ None of the included studies reported substantial reductions in dosage, or withdrawal of DAAs or immunosuppressive agents. Kwo et al^[36]^ studied 34 HCV-infected liver transplant recipients with minimal allograft fibrosis treated with DAAs for 24 weeks and reported no episodes of rejection, and only 15% of patients had anemia requiring erythropoietin therapy. Raschzok et al^[40]^ reported 70% SVR12 in their multi-center cohort of 40 HCV-infected liver transplant recipients treated for 24 weeks of DAAs, and there was no allograft loss or rejection during treatment; furthermore, there were no substantial dosing interactions of immunosuppression. Pungpapong et al^[41]^ found that 72% of 25 patients who received DAAs developed anemia that required intervention or dose reduction. Overall, unlike IFN-α, DAAs are effective cures for HCV infection and do not impair liver and renal function, and avoid acute rejection, allograft loss, and serious AEs.

Finally, there are some limitations in our study. First, it is a retrospective study lacking the characteristics of random grouping and high patient homogeneity between studies. Furthermore, this study was based on the data of a single race with a small sample size, which would lead to sampling errors. Therefore, large-scale, multiracial prospective studies are needed to prove the above conclusions. In addition, the length of the follow-up period was insufficient. Lastly, there are no available literature for evaluating the effectiveness and safety of using DAAs versus not using DAAs.

In conclusion, our meta-analysis explores the efficacy and safety of DAAs for treating HCV-infected RTRs, and suggests that DAAs can resolve HCV infection effectively within 24 weeks with significant improvement of liver function and without loss of allograft function. In addition, there are only a small number of serious AEs. The high efficacy and tolerability also hold great promise for patients with end-stage kidney disease who receive HCV-positive renal organs, which could reduce waitlist time and mortality. Meanwhile, further large-scale, well-designed studies should be conducted to confirm our findings.
